# Molecular docking, molecular dynamics, and *in vitro* studies reveal the potential of angiotensin II receptor blockers to inhibit the COVID-19 main protease

**DOI:** 10.1016/j.heliyon.2020.e05641

**Published:** 2020-12-03

**Authors:** Radwan Alnajjar, Ahmed Mostafa, Ahmed Kandeil, Ahmed A. Al-Karmalawy

**Affiliations:** a Department of Chemistry, Faculty of Science, University of Benghazi, Benghazi, Libya; b Department of Chemistry, University of Cape Town, Rondebosch, 7701, South Africa; c Center of Scientific Excellence for Influenza Viruses, National Research Centre, Dokki, 12622, Cairo, Egypt; d Department of Pharmaceutical Medicinal Chemistry, Faculty of Pharmacy, Horus University-Egypt, New Damietta, 34518, Egypt

**Keywords:** Drug repurposing, ARBs, COVID-19, Docking, MD, MM-GBSA, *In vitro* studies, Computer science, Physics, Materials science, Chemistry, Biological sciences

## Abstract

Drug repurposing is the most rapid and economic way nowadays to rapidly provide effective drugs for our pandemic coronavirus disease 2019 (COVID-19). It was a great debate about ARBs whether to be stopped or continued for patients using them especially at the beginning of the COVID-19 pandemic. In this study, we carried out a virtual screening for almost all members of ARBs (nine) against COVID-19 main protease. Molecular docking as one of the important computational techniques was performed in this work. Interestingly, the tested compounds showed variable binding affinities in the order of N3 inhibitor (**10**, docked) > Fimasartan (**8**) > Candesartan (**2**) > Olmesartan (**7**) > Azilsartan (**9**) > Eprosartan (**5**) > Valsartan (**3**) > Losartan (**1**) > Irbesartan (**6**) > Telmisartan (**4**). Moreover, Fimasartan (**8**), Candesartan (**2**), and Olmesartan (**7**) were additionally estimated through molecular dynamic simulations monitored via computing the binding free energy using MM-GBSA. The results are promising for rapidly repurposing such drugs (especially, Fimasartan (**8**) and Candesartan (**2**)) after further preclinical and clinical studies either alone or in combination with others for the treatment of COVID-19 virus especially known to cause vasodilatation (to prevent blood coagulation) and to reduce inflammation and fibrosis (to prevent pulmonary fibrosis), with well-known safety profiles. *In vitro*, the virtual findings were consistent with the experimental testing of four representative ARBs. Out of the tested compounds, Olmesartan (**7**) showed the most promising anti-SARS-CoV-2 activity (IC_50_ = 1.808 μM, and CC_50_ = 557.6 μM) with high selectivity index (308.4) against SARS-CoV-2 in Vero E6 cells. This work may clarify and approve not only the safety of ARBs used by a large group of patients worldwide but also their possible effectiveness against the COVID-19 virus either as a prophylactic or treatment option. It intended also to give a clear spot on the structure-activity relationship (SAR) required for the future design of new drugs targeting the newly emerged SARS-CoV-2 protease by medicinal chemists.

## 1 Introduction

By December 2019, a novel coronavirus, SARS-CoV-2, has been detected initially in China. The virus outbreak took place first in Wuhan city and continued to spread worldwide [1]. The world's attention has focused on the world out of our sight of viruses like never before, especially by causing 32,952,046 total confirmed cases till 26 September 2020 including 996,276 confirmed deaths all over 215 countries, areas, or territories as published officially on the World Health Organization website [2]. Being highly contagious, it has widely spread to every corner of the world [3].

Severe acute respiratory syndrome coronavirus 2 (SARS-CoV-2) is causing severe respiratory syndrome in humans [4]. Its main protease (M^pro^, 3CL^pro^) is approved to be an attractive drug target among coronaviruses, due to its very crucial role in controlling viral replication and transcription [5, 6, 7].

One of the most important and recent approaches to investigate the activity of a drug is the simulating nature through computational structure-based drug discovery. In this process, computer software test compounds into the selected binding sites in three-dimensional models of the protein targets. The interaction between the tested compounds and the binding site can be quantified using physics-based equations to calculate their binding affinities. The best compounds then tested experimentally on animal models to ensure their real binding and to confirm their effectiveness (such as stopping viral infectivity) [8].

Drug repurposing is the reuse of an existing drug for the treatment of a new disease that is outside the scope of the original intended or approved one [9]. It leads to fast drug reach at a lower cost and shorter time than *de novo* drug development [10, 11, 12]. The importance of drug repurposing is currently well-understood especially after the emergence of the pandemic COVID-19 [13, 14]. Drugs such as ivermectin, ribavirin, remdisivir, and sofosbuvir were tested *in silico* and *in vitro* for their potential as a treatment for COVID-19 [15].

However, most of the COVID-19 patients belong to elder stages with cardiovascular comorbidities such as hypertension, coronary artery disease, heart failure, or chronic kidney disease [16, 17, 18]. One of the most important classes for the treatment of such diseases are angiotensin-converting enzyme inhibitors (ACEIs) or angiotensin II receptor blockers (ARBs) [16].

**Figure 1:**
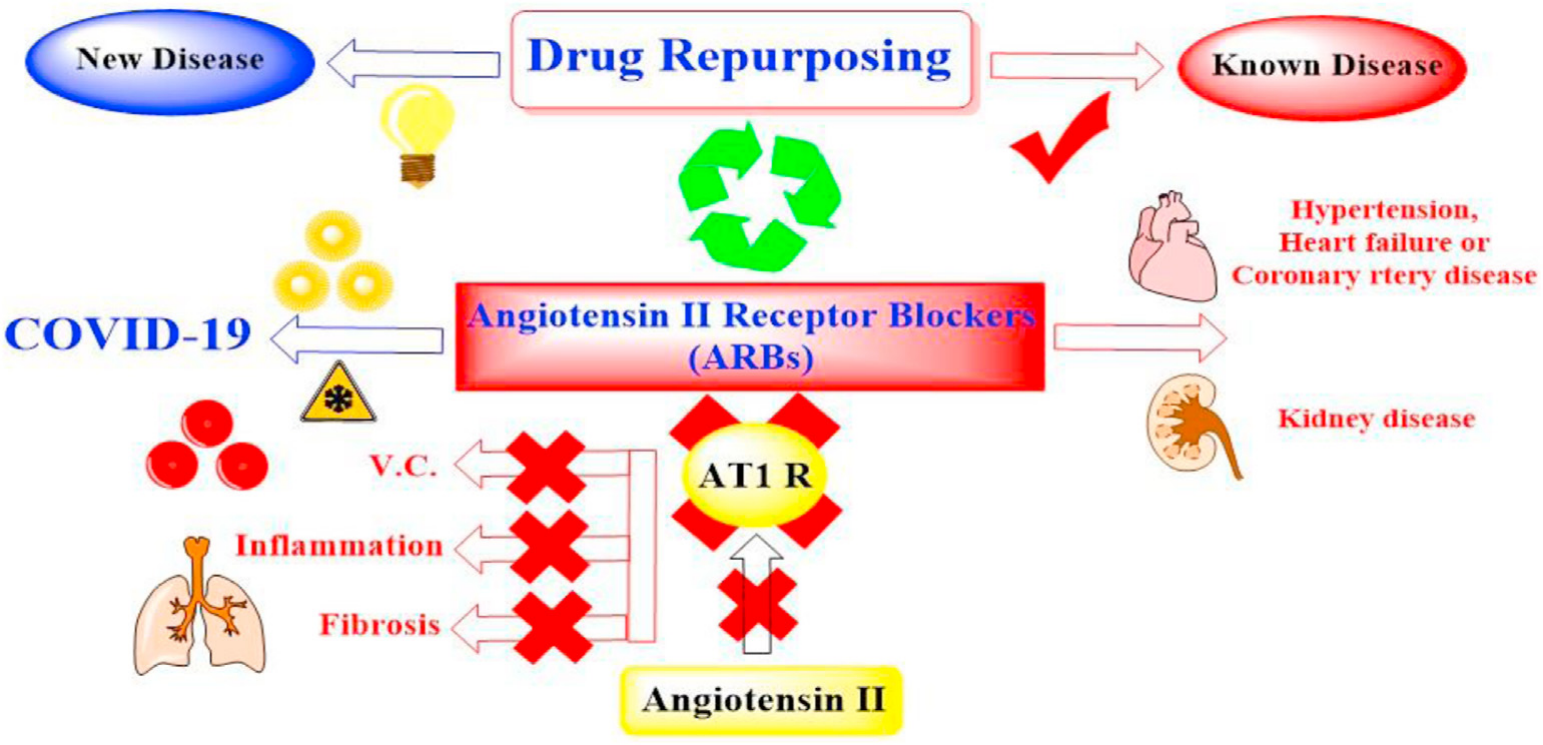
Drug repurposing of ARBs as COVID-19 inhibitors especially causing V.D. and preventing inflammation and fibrosis characteristic to pandemic infection.

Both ACEIs and ARBs have been approved to decrease the progression of pulmonary complications in susceptible patients and to decrease the risk of pneumonia [19]. Moreover, ARBs are known to antagonize the actions of angiotensin II by blocking AT1 receptors preventing vasoconstriction, apoptosis, and proinflammatory and fibrosis effects (Figure 1) [20].

Interestingly, Zhang et al. observed that hypertensive cases hospitalized with COVID-19, among them those treated with ACEI/ARB were at a lower observed risk of mortality compared with nonusers [21]. Furthermore, it was approved that COVID-19 patients were not affected by the use of the renin-angiotensin-aldosterone system (RAAS) inhibitors and so should not be stopped to prevent a progression of COVID-19 [22].

Depending on the previously mentioned therapeutic effects of ARBs as vasodilators (to decrease the tendency for coagulation), antiapoptotic, anti-inflammatory, and antifibrotic (to decrease the tendency for pneumonia) caused by COVID-19, hoping to repurpose them effectively for the potential treatment of pandemic COVID-19 infection.

**Figure 2:**
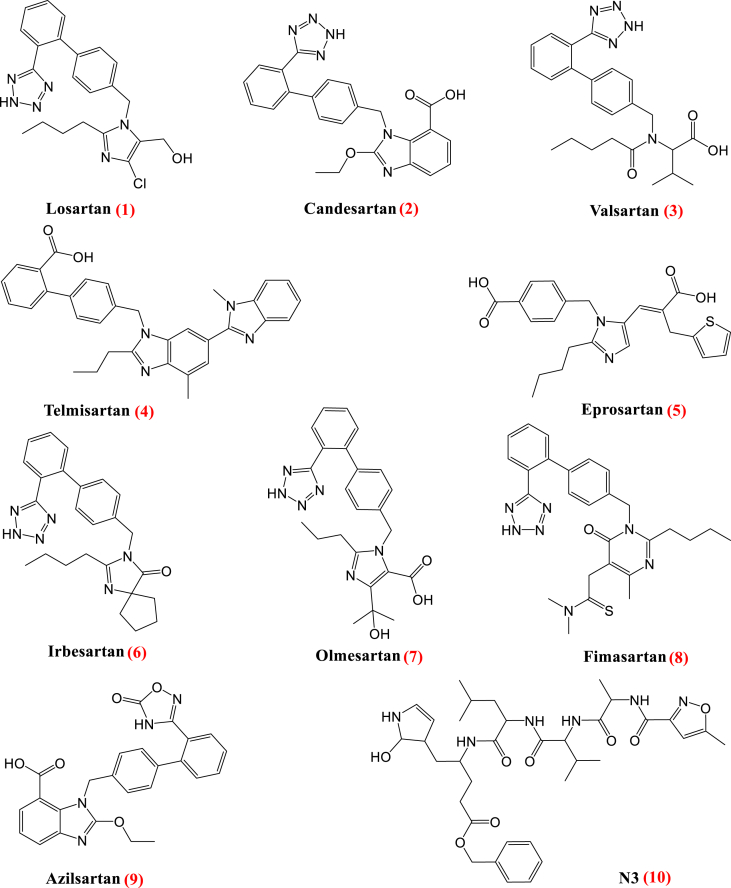
Chemical structures of Losartan **1**, Candesartan **2**, Valsartan **3**, Telmisartan **4**, Eprosartan **5**, Irbesartan **6**, Olmesartan **7**, Fimasartan **8**, Azilsartan **9** and N3 **10**.

In this study, angiotensin receptor blocker (ARB) drugs (Figure 2) were selected for molecular docking studies against M^pro^. Furthermore, molecular dynamic (MD) simulations were passed out on the best-docked drug-protein complexes to acquire more acceptance of the affinity between the ligands and the COVID-19 main protease active site in the frank solvent model for 150 ns to evaluate the stability of the ligands within the binding site of the protein. These ligand-protein complexes were follower to the Molecular Mechanics/Generalized Born and Surface Area (MM/GB-SA) calculations to evaluate the consistent relative binding free energies.

## 2 Materials and methods

Both docking studies using MOE 2019 suite [23] and molecular dynamic simulation studies using the Desmond simulation package of Schrödinger LLC [24] were performed to examine and confirm the binding affinities and modes of the FDA approved ARB drugs against COVID-19 main protease. The co-crystallized inhibitor (N3) was used as a standard reference.

### 2.1 Docking studies

#### 2.1.1 Preparation of the tested drugs

The tested drugs were obtained from the PubChem website. They were prepared according to the default method described before [25]. The database containing both the co-crystallized N3 inhibitor and the aforementioned drugs was formed and saved as an MDB file for docking.

#### 2.1.2 Preparation of SARS-CoV-2 main protease (M^pro^)

The Protein Data Bank website was used to download the crystal structure of the main protease (M^pro^) of SARS-CoV-2 (PDB code 6LU7) [26]. It was prepared following the same preparation steps described [25].

#### 2.1.3 Docking of the tested drugs to the viral M^pro^ binding pocket

At first, a validation process was done for the co-crystallized N3 and a valid behavior was confirmed by obtaining low RMSD values between the docked and co-crystallized ones [27, 28].

Docking of the aforementioned database containing the nine tested ARBs and the co-crystallized inhibitor N3 was performed according to the previously discussed procedure [29]. The best-obtained poses were selected according to their scores, binding modes, and rmsd_refine values.

### 2.2 Molecular dynamic simulation

The Molecular dynamic simulations were performed using the Schrödinger LLC package [24]. The NPT ensemble (T = 300 K and P = 1 bar) was performed. The length of the simulation was 150 ns and the relaxation time for all selected poses was one ps. The force field parameters (OPLS3) were applied [30]. A cutoff radius of 9.0 Å in Coulomb interactions, and orthorhombic periodic box boundaries were set away from the protein atoms at 10 Å. The water molecules were described using TIP3P model [31, 32]. Salt concentration was applied using Desmond System builder [33] and adjusted to 0.15 M NaCl. The pressure control was performed with a coupling constant of 2.0 ps using the Martyna−Tuckerman−Klein chain coupling scheme. The temperature control was applied through the Nosé−Hoover chain coupling scheme [34, 35]. All of the obtained data were analyzed and recorded according to the previously mentioned methods [36].

### 2.3 MD trajectory analysis and prime MM-GBSA calculations

To observing the interactions' influence in ligand-protein stability, Maestro software was used. The MM – GBSA was done to estimate ligand strain energies and the ligand binding free energies for docked molecules over the 150 ns period with thermal_mmgbsa.py python script delivered via Schrodinger which receipts a Desmond trajectory file, separates it into individual snapshots, runs the MMGBSA calculations on each frame, and yields the average calculated binding energy.

### 2.4 MTT cytotoxicity assay

To assess the half-maximal cytotoxic concentration (CC_50_), stock solutions of the tested ARBs were dissolved in 10 % DMSO in ddH_2_O and diluted further to the working solutions with DMEM. The cytotoxic activity was tested by applying the MTT method with minor modifications in VERO-E6 cells. Briefly, the cells were seeded in 96 well-plates and incubated at 37 °C and 5% CO_2_ for 24 h. After that, the cells were treated with different concentrations of the tested ARBs in triplicates. And then the total methodology was completed as previously mentioned in detail [37]. The concentration caused a 50% cytotoxicity (TC_50_) was obtained by plotting the% cytotoxicity versus sample concentration [38].

### 2.5 Inhibitory concentration 50 (IC_50_) determination

The IC_50_ concentrations were determined as previously described [39]. Briefly, in 96-well tissue culture plates, 2.4×10^4^ Vero-E6 cells were distributed in each well and incubated overnight at a humidified 37 °C incubator under 5% CO_2_ condition. The cell monolayers were then washed once with 1x PBS and subjected to virus adsorption for 1 h at room temperature (RT). The cell monolayers were further overlaid with 50 μl of DMEM containing varying concentrations of the test ARBs. Following incubation for 72 h, the cells were fixed for 20 min using 100 μl of 4% paraformaldehyde and stained using 0.1% crystal violet in distilled water for 15 min at RT. The crystal violet dye was then dissolved using 100 μl absolute methanol per well and the optical density of the color was measured using Anthos Zenyth 200rt plate reader at 570 nm. The IC_50_ of the compound is that required to decrease the virus-induced cytopathic effect (CPE) by 50%, compared to the virus control.

## 3 Results and discussion

### 3.1 Docking studies

The ligand-binding site of COVID-19 M^pro^ is located in the groove between a Cys–His catalytic dyad. The COVID-19 virus M^pro^ binding pocket is fitted with the N3 inhibitor composed of only one polypeptide and showing an asymmetric unit. Molecular docking of Losartan **1**, Candesartan **2**, Valsartan **3**, Telmisartan **4**, Eprosartan **5**, Irbesartan **6**, Olmesartan **7**, Fimasartan **8**, Azilsartan **9,** and N3 **10** into M^pro^ active site was performed. Their binding strength order was: N3 inhibitor (**10**, docked) > Fimasartan (**8**) > Candesartan (**2**) > Olmesartan (**7**) > Azilsartan (**9**) > Eprosartan (**5**) > Valsartan (**3**) > Losartan (**1**) > Irbesartan (**6**) > Telmisartan (**4**).

The selection of poses was done according to their better obtained binding scores and rmsd_refine values, especially most of them achieved very close binding modes compared to N3. The obtained scores, RMSD_Refine values, and interactions with M^pro^ pocket amino acids are represented in Table 1.

**Table 1:** Receptor interactions and binding energies of the identified ARB drugs and N3 inhibitor into the N3 inhibitor binding site of COVID-19 main protease.

No.	ARB drug	S^a^ kcal/mole	RMSD_Refine^b^	Amino acid bond	Distance A֯
1	Losartan	-7.58	0.98	Glu 166/H-acceptor His 163/H-donor Gln189/H-pi	2.95 3.00 3.50
2	Candesartan	-7.79	1.46	His163/H-donor His163/H-donor Glu166/H-acceptor Gln189/H-pi Gln189/H-pi	3.06 3.31 3.33 4.17 4.51
3	Valsartan	-7.83	1.23	Cys145/H-donor Thr26/H-acceptor Glu166/H-pi	2.89 2.98 4.04
4	Telmisartan	-8.16	1.77	Thr26/H-acceptor Glu166/H-pi Glu166/H-pi His41/pi-H	2.92 3.69 3.90 4.14
5	Eprosartan	-7.30	2.22	Gln189/H-acceptor Cys145/H-acceptor Met165/H-acceptor His41/pi-H	3.15 3.43 3.71 3.95
6	Irbesartan	-7.26	1.15	Thr26/H-acceptor Thr26/H-donor Thr25/H-pi	3.02 3.37 4.54
7	Olmesartan	-7.67	1.39	Gly143/H-donor Cys145/H-donor His41/H-donor His41/pi-H Glu166/H-pi	2.95 3.23 3.28 3.25 4.68
8	Fimasartan	-7.82	1.33	Gly143/H-donor Cys145/H-donor Asn142/H-donor Met165/H-donor Met165/H-acceptor Gln189/H-pi	3.21 4.00 4.14 3.49 3.37 3.70
9	Azilsartan	-7.92	1.59	His163/H-donor His164/H-acceptor Asn142/H-acceptor Asn142/H-pi Gln189/H-pi	3.09 3.36 3.40 4.02 3.52
10	N3	-10.16	1.92	Asn142/H- acceptor Phe140/H-acceptor Gln189/H-acceptor Cys145/H-acceptor His41/pi-H	2.78 3.29 3.48 3.74 4.18

a **S**: the score of a compound placement inside the protein binding pocket.

b **RMSD_Refine**: the root-mean-squared-deviation (RMSD) between the predicted pose and those of the crystal one (after and before refinement process, respectively).

The docked N3 (**10**) inside the COVID-19 virus M^pro^ pocket achieved a binding score of -10.16 kcal/mol beside the formation of four H-bonds with Asn142, Phe140, Gln189, and Cys145, and one H-pi bond with His41amino acids of protease. On the other hand, it was found that especially, Fimasartan (**8**), Candesartan (**2**), and Olmesartan (**7**) members of ARBs having very close binding modes relative to the N3 inhibitor.

Fimasartan (**8**) showed a binding score of -7.82 kcal/mol with the formation of five H-bonds with Gly143, Cys145, Asn142, and Met165, and one pi-H bond with Gln189 amino acids. Moreover, Candesartan (**2**) showed a binding score of -7.79 kcal/mol with the formation of three H-bonds with His163 and Glu166, one pi-H bond, and one H-pi bond with Gln189 amino acids. Furthermore, Olmesartan (**7**) showed a binding score of -7.67 kcal/mol with the formation of three H-bonds with Gly143, Cys145, and His41, one H-pi bond with His41, and one pi-H bond with Glu166 amino acids (Table 2). To compare the binding modes of ARBs and N3 inhibitor against COVID-19 main protease, 3D representations and surface and maps for each studied pose were introduced. The docking information of all tested compounds is represented in the supplementary material.

**Table 2:** The 3D binding interactions and the 3D positioning of the best-docked ARBs (**8**, **2**, &**7**) and N3-binding pocket within COVID-19 main protease (PDB: 6LU7) compared to the N3 (Docked).

Drug	3D interaction	3D protein positioning
Fimasartan (**8**)		
Candesartan (**2**)		
Olmesartan (**7**)		
N3 (**10**)		

H-bonds are represented by red dashed lines while H-pi interactions by black dashed lines.

Briefly, the docking results of ARB drugs to M^pro^ of COVID-19 compared to its N3 inhibitor clarified greatly the binding modes of them. Some gave ideal binding modes indicating high affinity and predicted intrinsic activity as well.

### 3.2 Molecular dynamics (MD) simulation

Docking protocols are usually rapid and imprecise—however, docking deficiencies protein flexibility, which may broker with the accuracy of the consequential ligand-protein complexes. So, further computationally expensive but additional accurate molecular dynamic simulation techniques might improve a better complementary with docking. Generally, MD simulation is used to estimate the macromolecule manners, and it depends on traditional mechanics and using Newton's equation of motion to compute the speed of and position of each atom of the considered system. Thereby, MD performs a more intensive conformational examination than docking does, which gives a more accurate illustration of protein motion. Taking into attention, the stated facts, MD simulations were proceeded using the Desmond package on the ligand-potential complex to mimic the interaction of these drugs with COVID-19 main protease active site for 150 ns. Fimasartan (**8**), Candesartan (**2**), and Olmesartan (**7**) compounds were further selected for MD simulations (Figure 2).

### 3.3 Protein and ligand RMSD analysis

**Figure 3:**
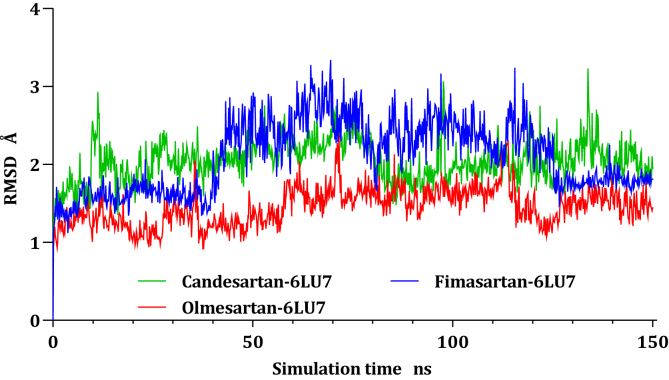
Plots of RMSD for Cα atoms (Å) concerning the initial structure vs simulation time (ns) for all the complexes.

RMSD values of Cα atoms for all the complexes were estimated concerning their initial structure, attempting to record the effect of the compounds on the conformational stability of 6LU7 during simulations, the results were plotted in (Figure 3) as a function of the simulations time. The Candesartan-6LU7 complex reach equilibrium since the start of the calculations around ten ns with side chains residuals fluctuate from time to time, even though the fluctuation was within 1 Å, where the Fimasartan-6LU7 complex was stable till 40 ns before it starts fluctuated at 40 ns till reach equilibrium again at 130 ns, this fluctuation is due to the movement of the unfolded side chain, where Ser1 break bond with Gln306 to form a new interactions with Ser284 and Glu290 led to stability at the 130 ns. The Olmesartan-6LU7 complex exhibits stability during all trajectories; it fluctuated within 1 Å.

**Figure 4:**
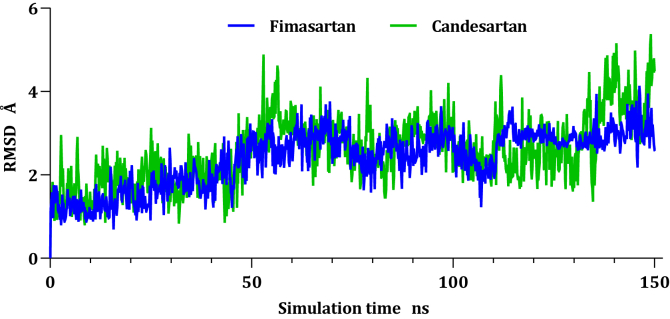
Plots of RMSD for ligand atoms (Å) concerning the initial structure vs simulation time (ns) of all the complexes.

The RMSD of a ligand that is aligned and measured just on its reference conformation within the active site was represented by plotting the RMSD of ligands as a function of simulation time (Figure 4), Fimasartan (**8**) and Candesartan (**2**) move around 4 Å concerning their reference position within the active site before reach equilibrium at 50 ns.

**Figure 5:**
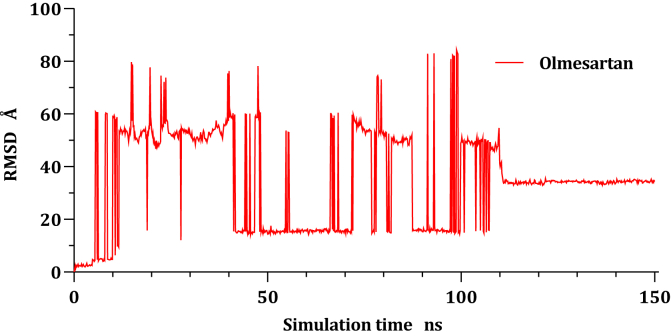
Plots of RMSD for Olmesartan atoms (Å) concerning the initial structure vs simulation time (ns) for all the complexes.

**Figure 6:**
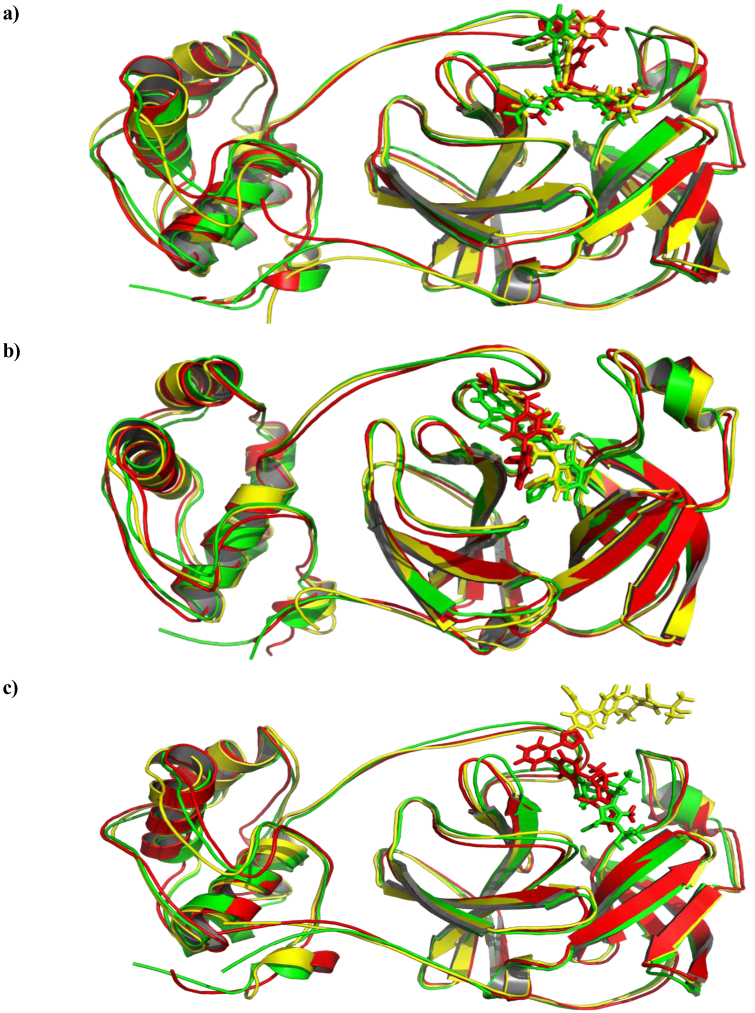
The aligned structures of Ligands-6LU7 during simulation; green 0 ns, yellow 75 ns, red 150 ns.

Olmesartan (**7**) was unstable within the active site of the COVID-19 main protease the drug shifted around protein during whole trajectories before reaching equilibrium at around 110 ns and settle down at a new site which is 40 Å far away from its original location as it can be seen in Figure 5. Moreover, Figure 6a, **6b**, and **6c** show the ligand-protein alignment during simulations time for Olmesartan-6LU7, Fimasartan-6LU7, and Candesartan-6LU7, respectively.

**Figure 7:**
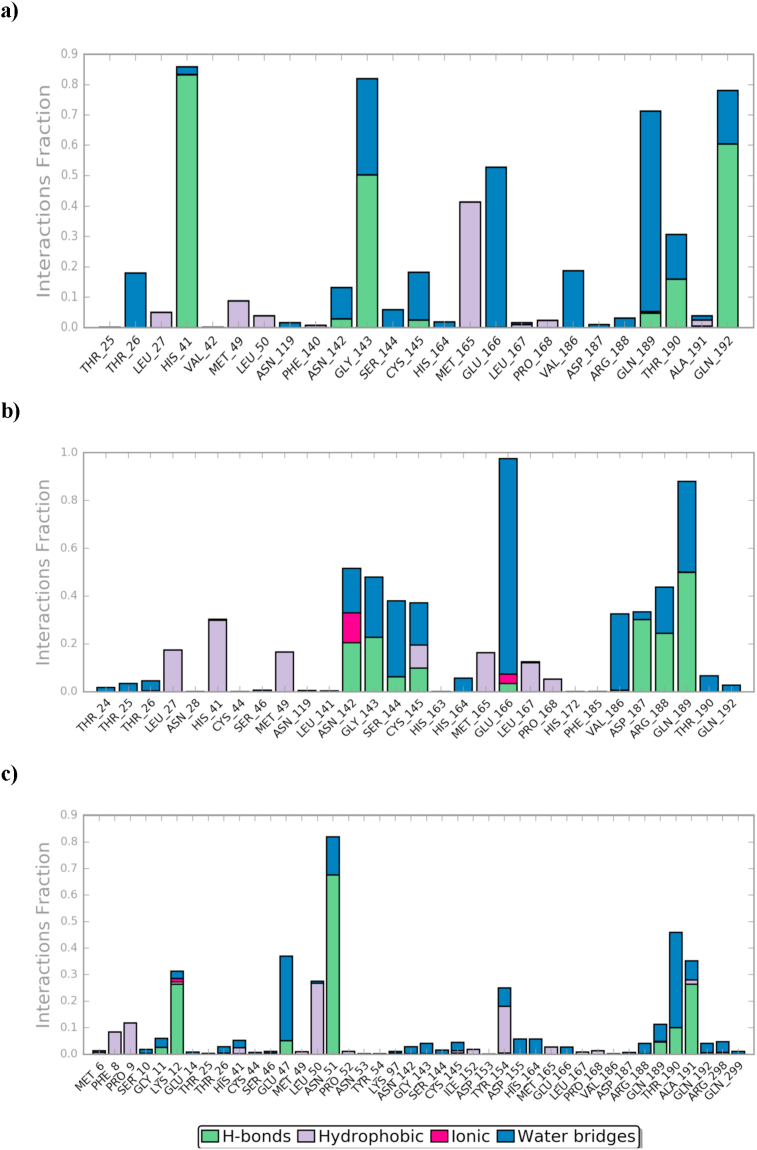
The histogram of **a)** Fimasartan-6LU7, **b)** Candesartan-6LU7, and **c)** Olmesartan-6LU7 contact throughout the trajectory.

**Figure 8:**
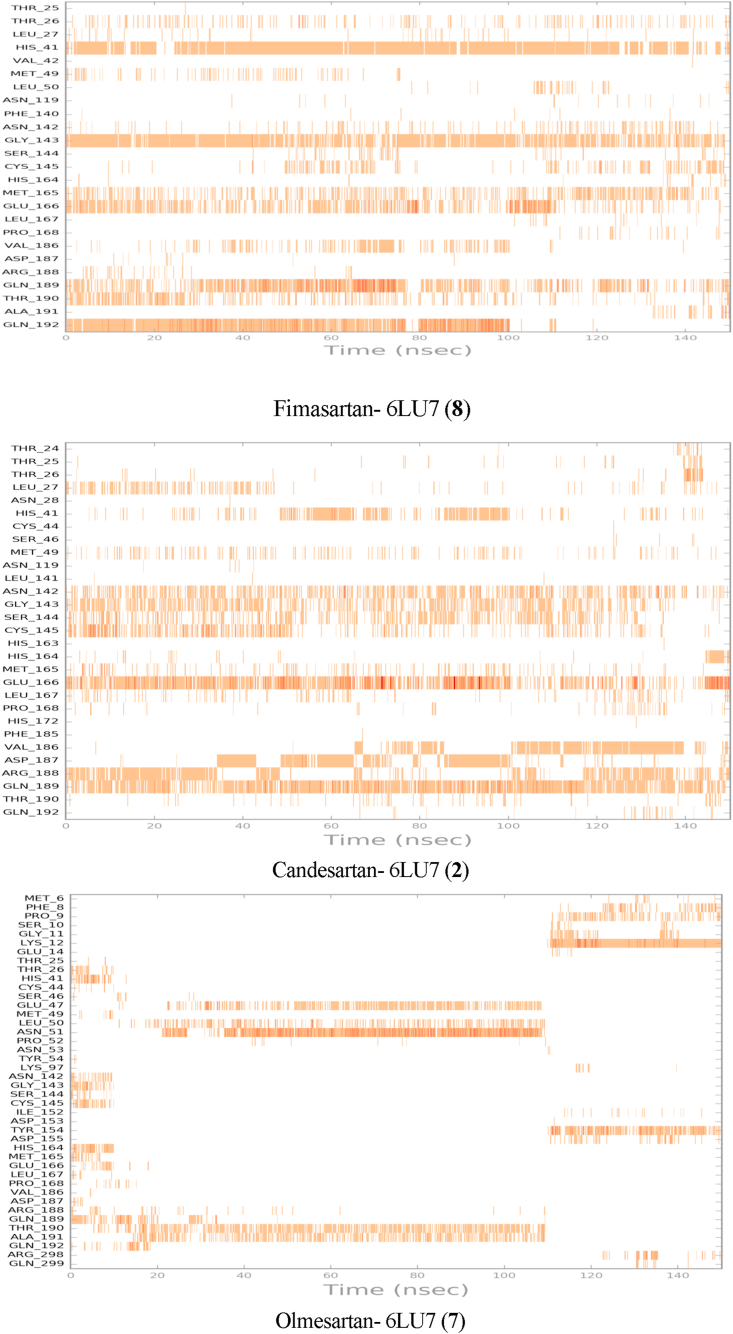
Fimasartan- 6LU7, Candesartan- 6LU7, and Olmesartan - 6LU7 interactions shown in each trajectory frame by the active site amino acids, zero interaction are represented by **white** while more interactions by the **deep color**.

The active site contains the following polar amino acid threonine (Thr190), glutamine (Gln143, Gln189, and Gln192), nonpolar amino acid methionine (Met49, Met165) and leucine (Leu27, Leu50), positively charged amino acid histidine (His41), and negatively charged amino acid glutamic (Glu166). As it can be seen from Figure 7 and Figure 8, which were generated with simulation interactions, diagram panel implemented in Maestro software, these histograms explain the contacts that occur during the simulations between ligands and protein.

In the case of Fimasartan (**8**), His41, Gln192, and Gly143 were able to hold down the hydrogen bonding contacts during 85 %, 65 %, and 52 % of the time, respectively. Water bridge hydrogen bonding where one water used as a bridge between ligand and protein was also formed with Glu166, Gln189, and Gly143 residuals and Fimasartan (**8**), finally, only one hydrophobic interaction was formed with Met165 during 35 % of simulation time.

Candesartan (**2**) formed a more but weaker hydrogen bonding the strongest interaction was with Gln189 which was 50 % of the time, where interactions with Asp187, Arg188, Gly143, Asn142, Cys145, and Ser144 were around 10–30 % of the time, which led to higher MM-GBSA binding energy (Table 3), hydrophobic attractions were formed with His41, Leu27, and Met49 during 30 %, 18 %, and 16% of trajectories, respectively. A small and neglectable ionic interaction occurs with Asn142 and Glu166 less than 10 % of the time.

**Table 3:** Prime MM-GBSA energies for ligands binding at the active site of COVID-19 main protease compared to N3 inhibitor.

	ΔG Binding	Coulomb	Covalent		H-bond	Bind Packing	Lipo	Solv_GB	vdW
Fimasartan (**8**)	-50.33	-15.69	3.03	-1.43		-14.42	-1.68	34.82	-54.95
Candesartan (**2**)	-53.05	9.17	1.92	-1.71		-15.23	-3.41	3.30	-47.10
Olmesartan (**7**)	-35.21	-3.64	1.88	-1.58		-12.96	-0.92	9.63	-27.62
N3 (**10**)	-88.18	-29.70	2.44	-2.41		-17.55	-1.10	28.82	-68.68

Olmesartan (**7**), on the other hand, was not stable within the active site, it was able to maintain hydrogen bond with the following residuals Asn51, Thr190, and Ala191 up to 110 ns before it lost these interactions, which led to moving out of the active site. Olmesartan later established a new hydrogen bonding with Lys12 and hydrophobic attraction with Tyr154.

### 3.4 MM-GBSA study

To calculate the average binding energy for equilibrated MD trajectory, further analysis using 200 selected snapshots with a 50 ps interval. The equation used to calculate the binding energy:

$$\Delta {\text{G}}_{\text{bind}}=\Delta {\text{E}}_{\text{MM}}+\Delta {\text{G}}_{\text{solv }}+\Delta {\text{G}}_{\text{SA}}$$

where ΔE_MM_ is the difference in minimized energies as following:

$$\Delta {\text{E}}_{\text{MM}}={\text{E}}_{\left(\text{complex}\right)}-{\text{E}}_{\left(\text{ligand}\right)}-{\text{E}}_{\left(\text{receptor}\right)}$$

The difference in GBSA solvation energy of the complex and the sum of ligand and protein solvation energies is denoted by ΔG_solv_. Also, ΔG_SA_ is the difference in surface area energy of the complex and the sum of protein and ligand.

The thermal_mmgbsa.py python script introduced by Schrodinger was used to calculate the average MM-GBSA binding energy which also generates Coulomb energy (Coulomb), covalent binding energy (Covalent), Van der Waals energy (vdW), lipophilic energy (Lipo), Generalized Born electrostatic solvation energy (Solv_GB), and Hydrogen-bonding energy (H-bond). All the obtained results are shown in Table 3.

From the MM-GBSA results, the most favored binding energy was exhibited by Candesartan (**2**) with strong vdW interactions and lipophilic energy (Table 3). In contrast, unfavored Coulomb energy was exerted by Candesartan which may result from the repealing with Glu166.

### 3.5 Experimental validation

**Figure 9:**
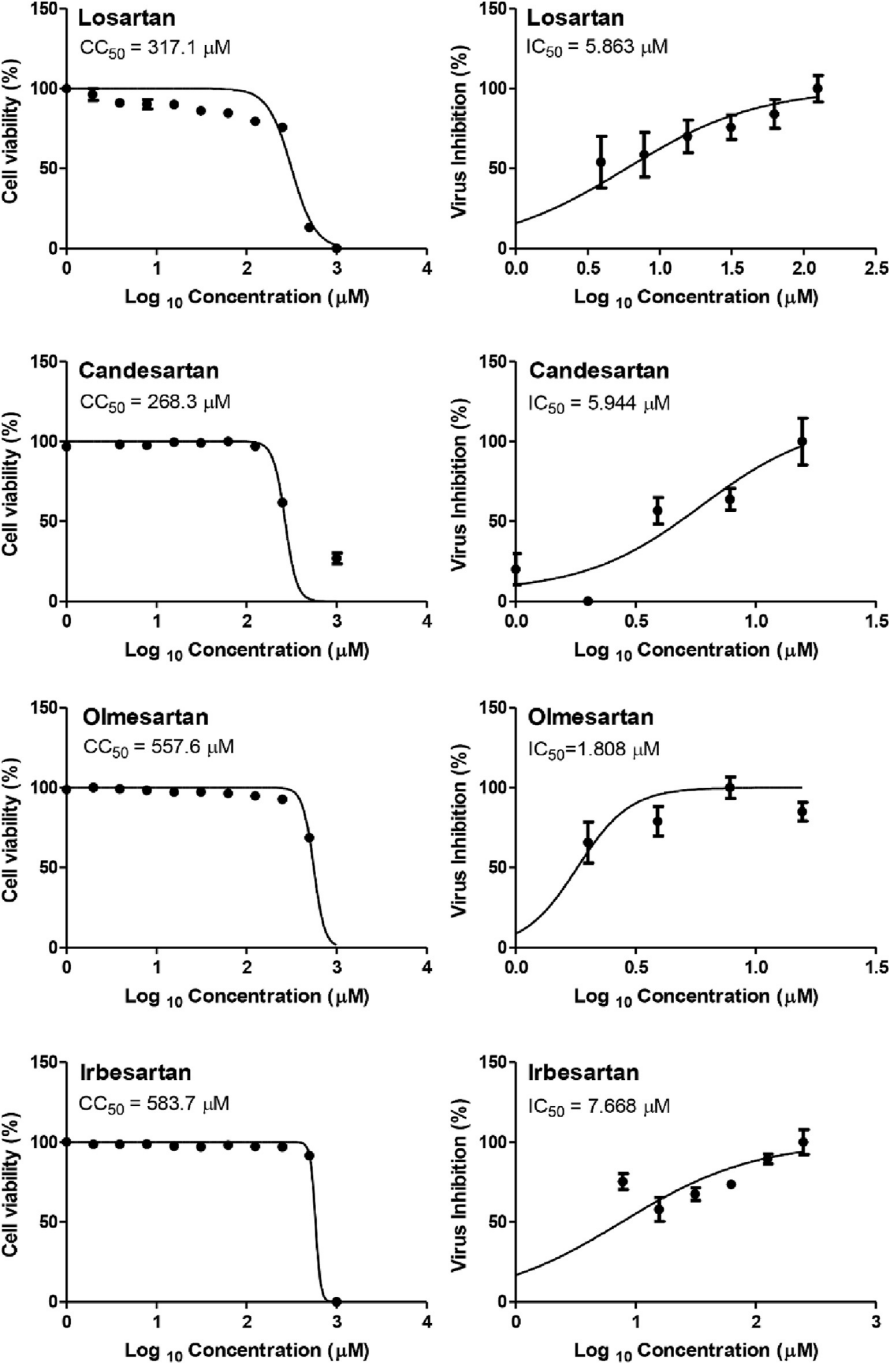
Dose-response curves for the tested drugs in Vero-E6 cells. Various dilutions of the drugs were applied to the 90% confluent cell monolayers and assayed after 72 h to determine the CC_50_ (half-maximal cytotoxic concentrations) or IC_50_ (half-maximal inhibitory concentrations). Nonlinear regression analysis of GraphPad Prism software (version 5.01) was used to calculate CC_50_ and IC_50_ by plotting log inhibitor versus normalized response (variable slope).

To validate the docking results which ordered the ARBs according to their binding affinities in the order of N3 inhibitor (**10**, docked) > Fimasartan (**8**) > Candesartan (**2**) > Olmesartan (**7**) > Azilsartan (**9**) > Eprosartan (**5**) > Valsartan (**3**) > Losartan (**1**) > Irbesartan (**6**) > Telmisartan (**4**) and which were confirmed further by molecular dynamic simulations, CC_50_ and IC_50_ were determined for Candesartan Cilexetil (prodrug of **2**), Olmesartan (**7**), Losartan (**1**), and Irbesartan (**6**). All tested compounds showed high to moderate antiviral activity against SARS-CoV-2 ranging from 1.808 to 7.668 μM (Figure 9). Collectively, the *in vitro* results of the tested compounds confirmed greatly the aforementioned computational studies except for Candesartan Cilexetil which is the available prodrug form of Candesartan (**2**) - (inactive *in vitro*)- and which explains clearly its deviation from our previous order. Interestingly, Olmesartan (**7**) showed the best selectivity index (>300) against SARS-CoV-2 in VERO E6 cells (Table 4).

**Table 4:** Selectivity indices of the tested ARBs.

Drug	CC_50_	IC_50_	Selectivity index (SI)
Candesartan Cilexetil (prodrug of 2)	268.3	5.944	45.1
Olmesartan (7)	557.6	1.808	308.4
Losartan (1)	317.1	5.863	54.1
Irbesartan (6)	583.7	7.668	76.1

**Abbreviations:** “CC_50_” half-maximal cytotoxic concentration; “IC_50_” half maximal inhibitory concentration; “SI” Safety index.

## 4 Conclusion

Nine ARB drugs widely used for the treatment of hypertension, coronary artery disease, heart failure, or kidney disease were subjected to molecular docking against COVID-19 main protease. The tested drugs exhibited variable degrees of affinities toward COVID-19 protease compared to N3 inhibitor in the order of N3 inhibitor (**10**, docked) > Fimasartan (**8**) > Candesartan (**2**) > Olmesartan (**7**) > Azilsartan (**9**) > Eprosartan (**5**) > Valsartan (**3**) > Losartan (**1**) > Irbesartan (**6**) > Telmisartan (**4**). The molecular dynamic simulations presented a moderate interaction between Fimasartan (**8**) and Candesartan (**2**) with the COVID-19 main protease with the latter being more favored; also, MD showed that Olmesartan (**7**) was not stable at all within the active site and left after 110 ns. The MM-GBSA binding energy showed that both Fimasartan (**8**) and Candesartan (**2**) had low binding energy compared to the N3 inhibitor by almost ~ -35 kcal/mol. In conclusion, it looks like-charged molecules are not favorable for the main protease active site of COVID-19. The CC_50_ and IC_50_ concentrations were determined for Candesartan Cilexetil (prodrug of **2**), Olmesartan (**7**), Losartan (**1**), and Irbesartan (**6**). All the tested compounds showed high to moderate antiviral activity against SARS-CoV-2 ranging from 1.808 to 7.668 μM. Interestingly, Olmesartan (**7**) showed the best CC_50_ and IC_50_ values (557.6 and 1.808 μM, respectively), and selectivity index (>300) against SARS-CoV-2 in VERO E6 cells. Finally, the present study confirmed the affinities of the tested ARB drugs against COVID-19 main protease. Such a drug especially, Fimasartan (**8**), Candesartan (**2**), and Olmesartan (**7**) members of ARBs are recommended to be further tested preclinically and clinically for proposed activity against COVID-19. They may be tested either alone or in combination. This work may clarify and approve not only the safety of ARBs used by a large group of patients worldwide but also its possible effectiveness against the COVID-19 virus either as a prophylactic or treatment option. Besides, the results may clarify greatly the SAR required for M^pro^ targeting, and facilitate the introduction of future new effective candidates against COVID-19.

## Declarations

### Author contribution statement

A. Al-Karmalawy: Conceived and designed the experiments; Performed the experiments; Analyzed and interpreted the data; Contributed reagents, materials, analysis tools or data; Wrote the paper.

R. Alnajjar, A. Mostafa and A. Kandeil: Performed the experiments; Analyzed and interpreted the data; Contributed reagents, materials, analysis tools or data.

### Funding statement

This research did not receive any specific grant from funding agencies in the public, commercial, or not-for-profit sectors.

### Declaration of interests statement

The authors declare no conflict of interest.

### Additional information

No additional information is available for this paper.

## Appendix A Supplementary data

6 Supplementary data_spl_ ARBs
