# Supplementary material for: Molecular docking, molecular dynamics, and in vitro studies reveal the potential of angiotensin II receptor blockers to inhibit the COVID-19 main protease
Source: Heliyon. 2020 Dec 3;6(12):e05641. doi: 10.1016/j.heliyon.2020.e05641 (PMC7713577; doi:10.1016/j.heliyon.2020.e05641)
Supplement: Supplementary file 1 — 6 Supplementary data_spl_ ARBs [file mmc1.pdf]

Supplementary data

Fig. 1: 2D and 3D docking representations of ARBs and N3 inhibitor against the binding site of the COVID-19 main protease.

<table>
<tr><th>No.</th><th>ARB drug</th><th>2D</th><th>3D</th></tr>
<tr><td></td><td></td><td>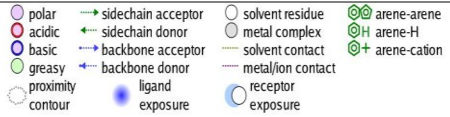
</td><td>Red dashed lines refer to hydrogen bonds, while the black ones denote hydrophobic interactions.</td></tr>
<tr><td>1</td><td>Losartan</td><td>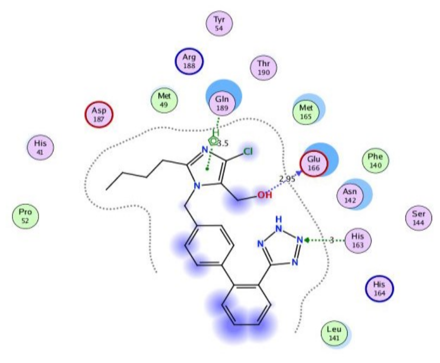</td><td>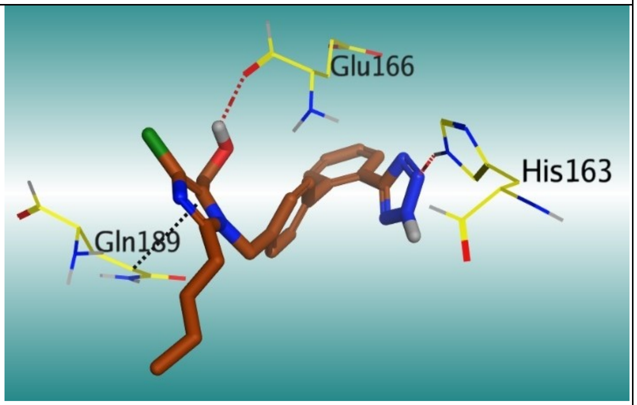
</td></tr>
<tr><td>2</td><td>Candesartan</td><td>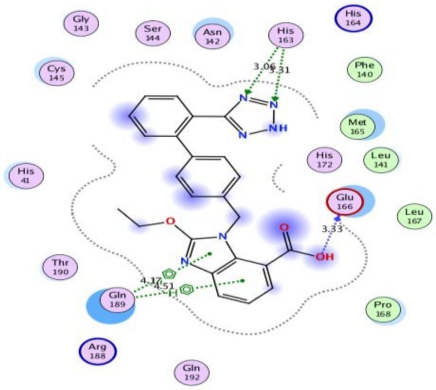</td><td>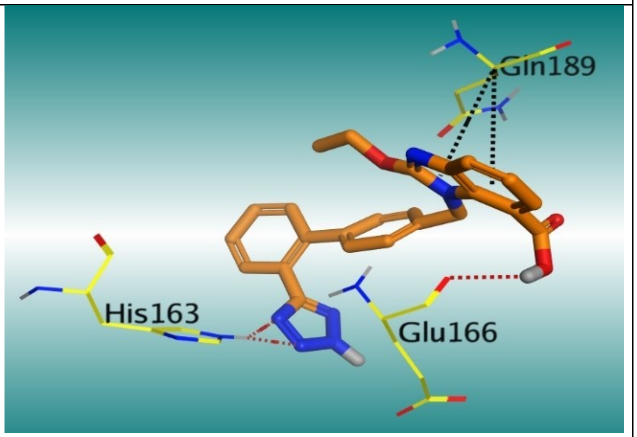
</td></tr>
<tr><td>3</td><td>Valsartan</td><td>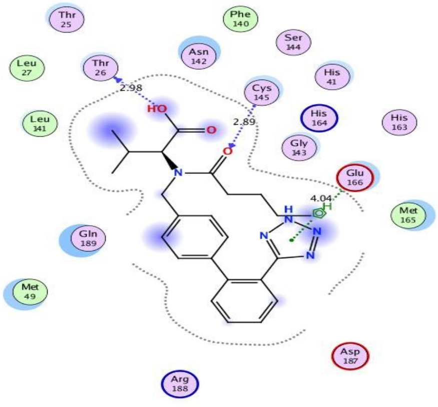</td><td>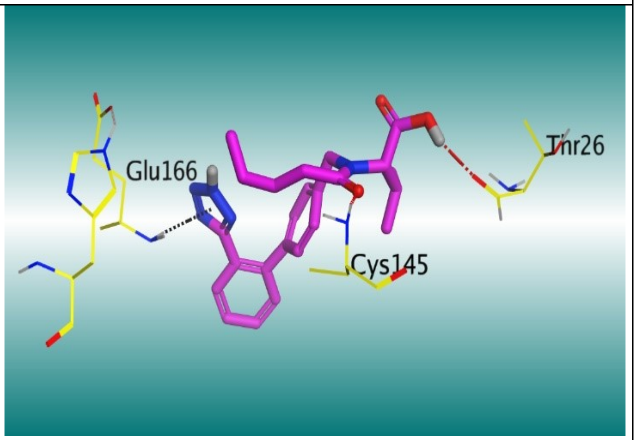
</td></tr>
</table>

<table>
<tr><td>4</td><td>Telmisartan</td><td>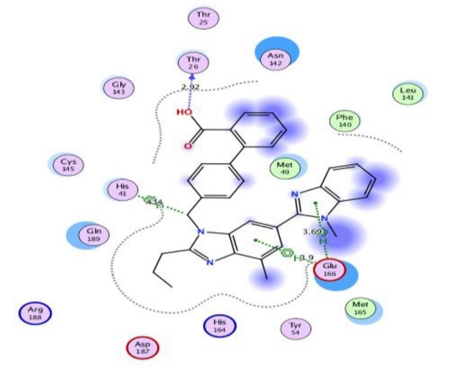</td><td>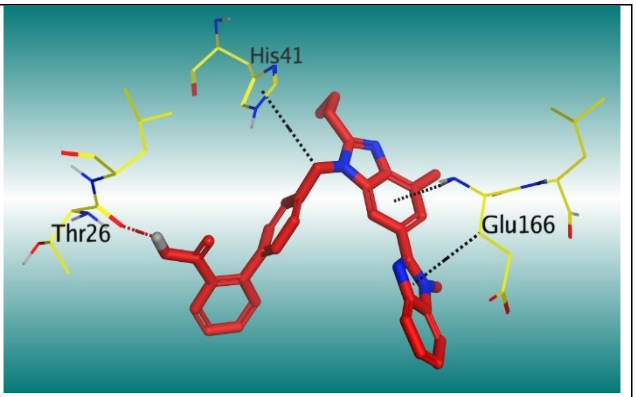
</td></tr>
<tr><td>5</td><td>Eprosartan</td><td>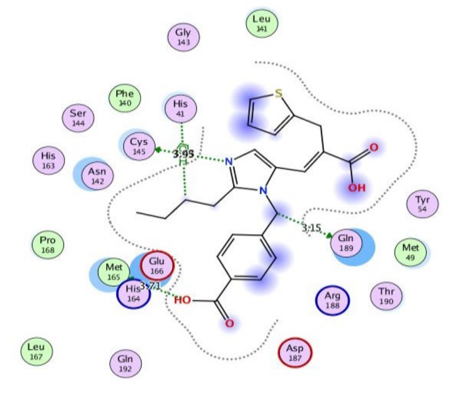</td><td>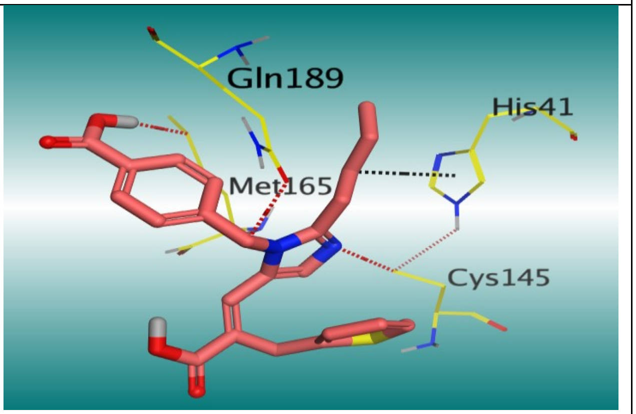
</td></tr>
<tr><td>6</td><td>Irbesartan</td><td>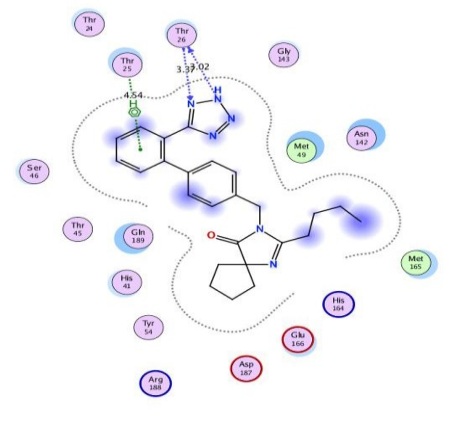</td><td>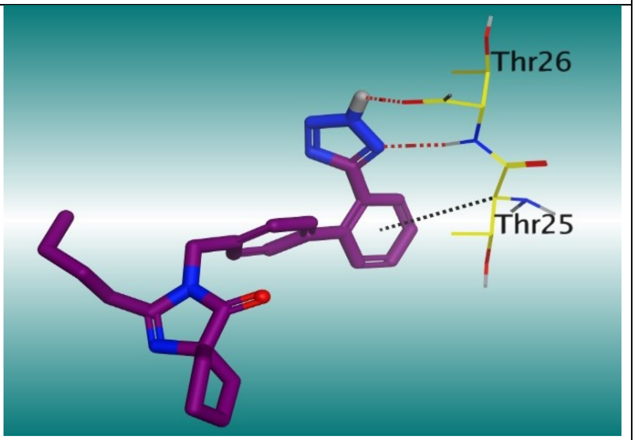
</td></tr>
<tr><td>7</td><td>Olmesartan</td><td>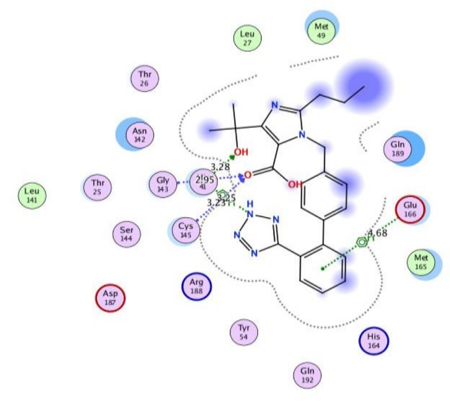</td><td>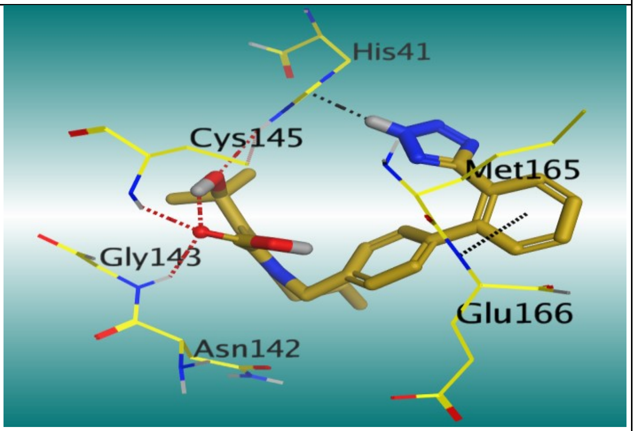
</td></tr>
</table>

**8**	Fimasartan	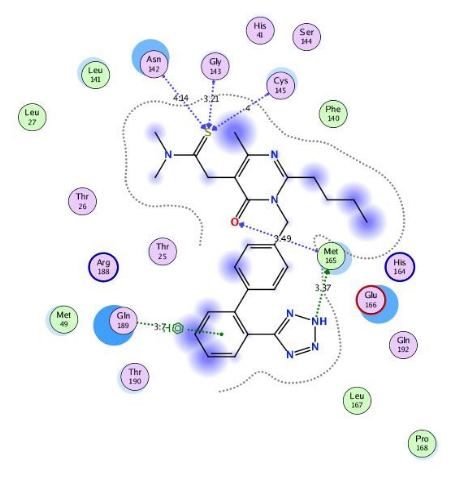	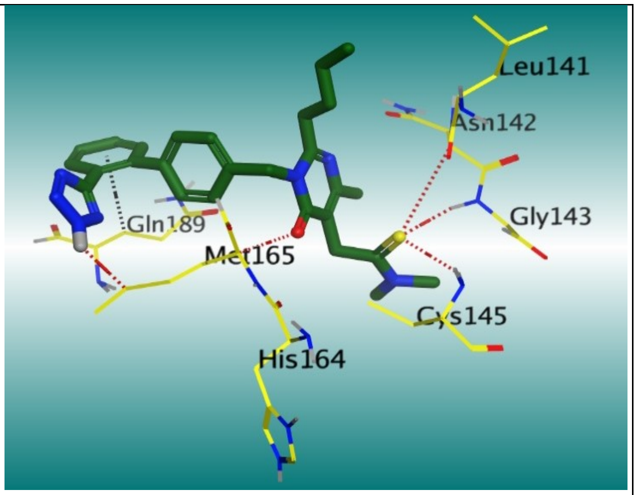
**9**	Azilsartan	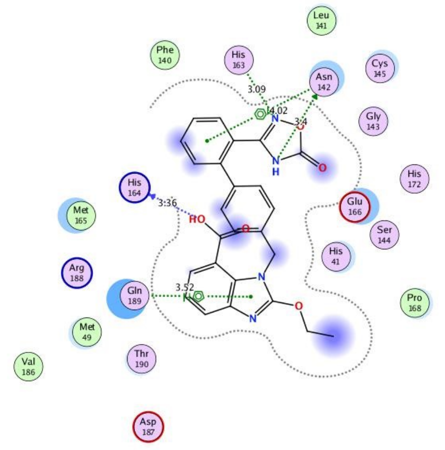	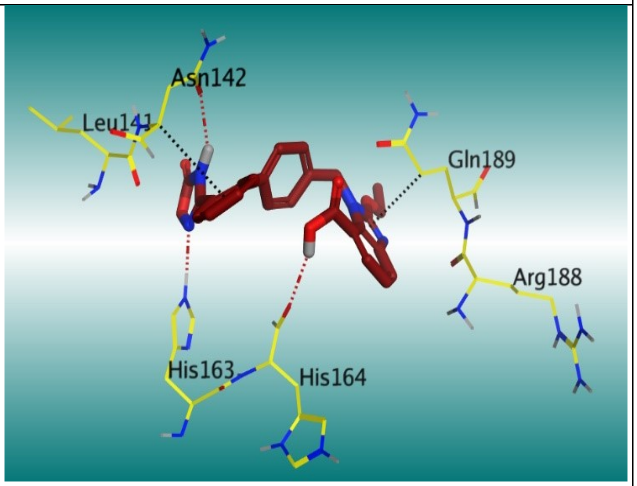
**10**	N3	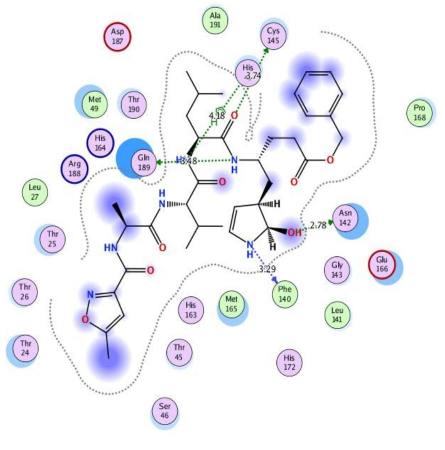	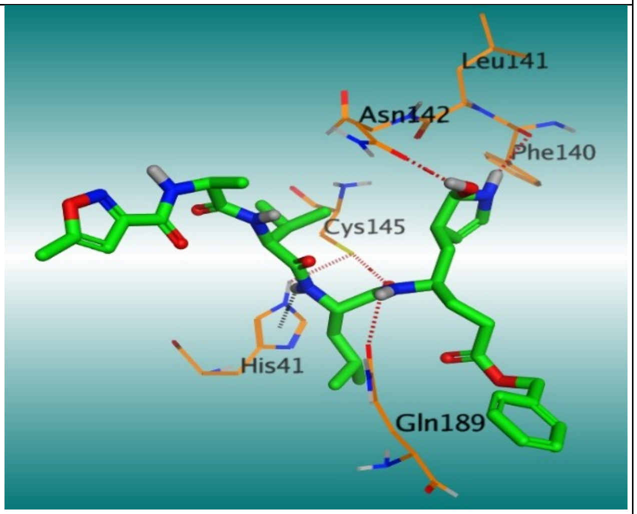

Fig. 2: **A)** Surface of M^{pro} showing the positioning and fitting of tested compounds, **B**) surface and maps of the tested compounds compared to N3 inhibitor

No.	ARB drug	A	B
**1**	Losartan	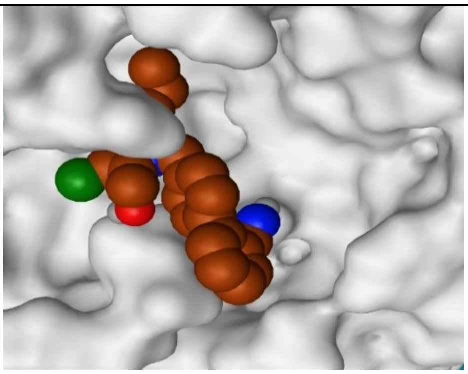	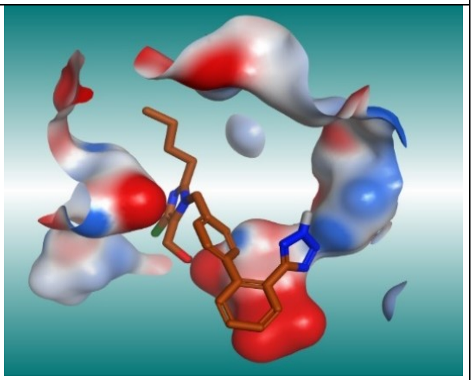
**2**	Candesartan	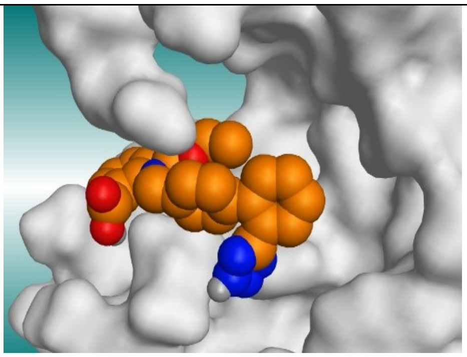	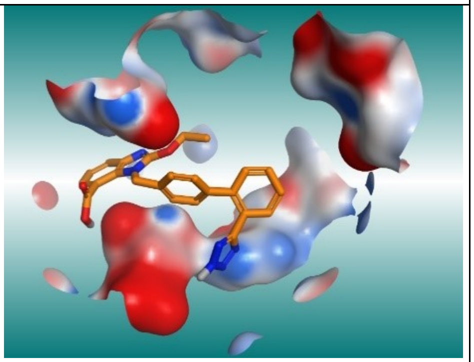
**3**	Valsartan	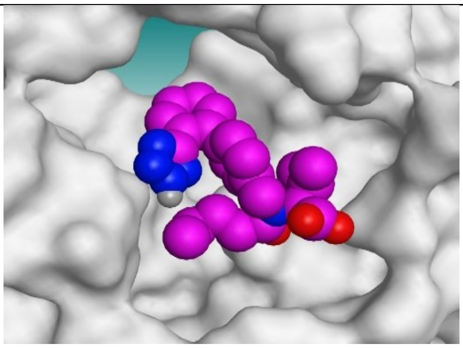	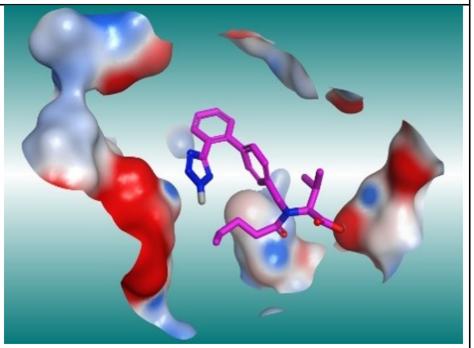
**4**	Telmisartan	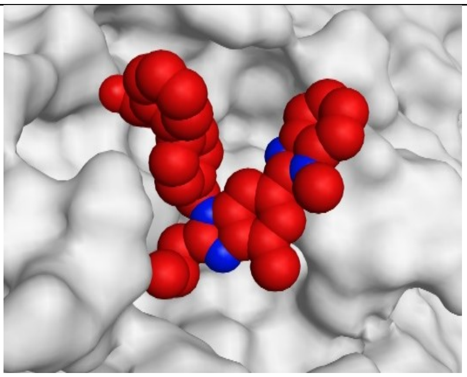	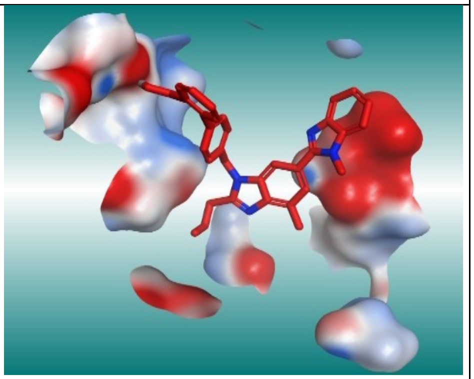

**5**	Eprosartan	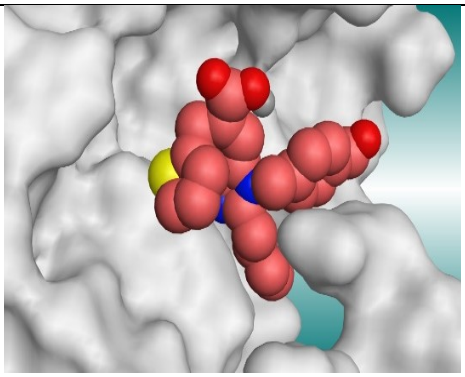	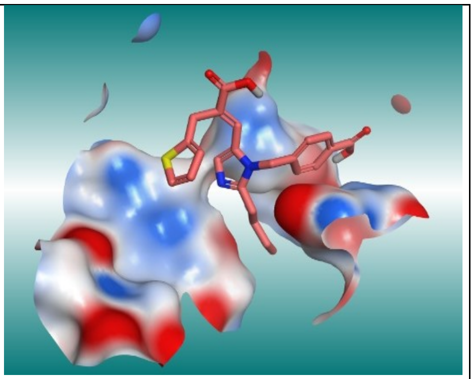
**6**	Irbesartan	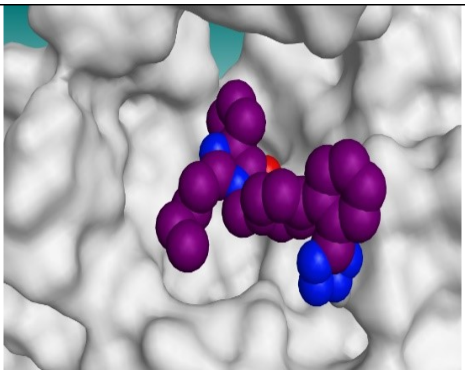	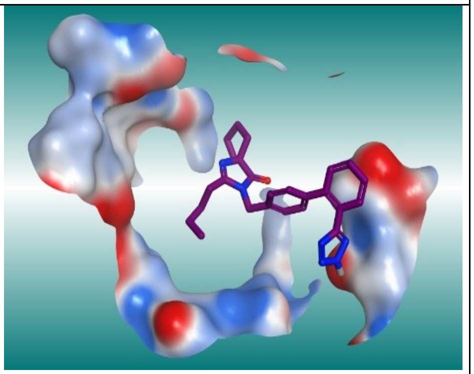
**7**	Olmesartan	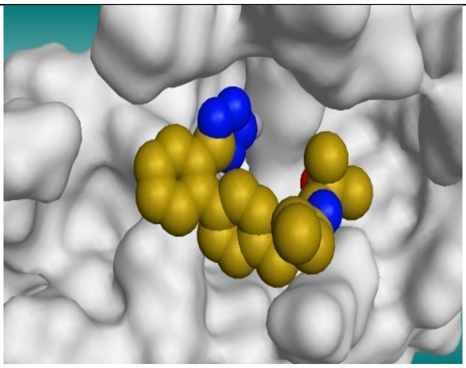	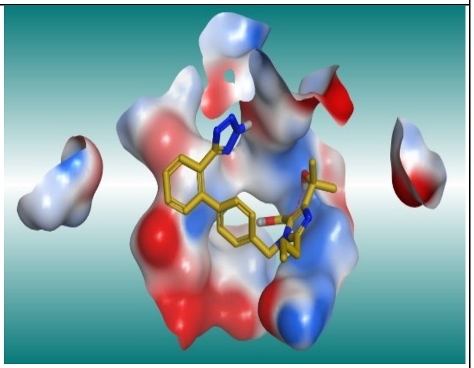
**8**	Fimasartan	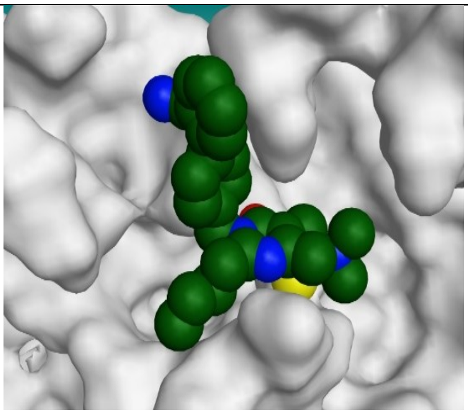	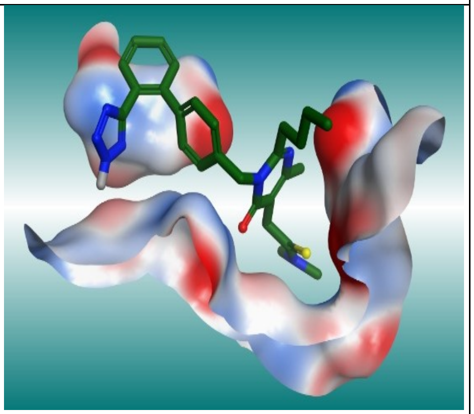

**9**	Azilsartan	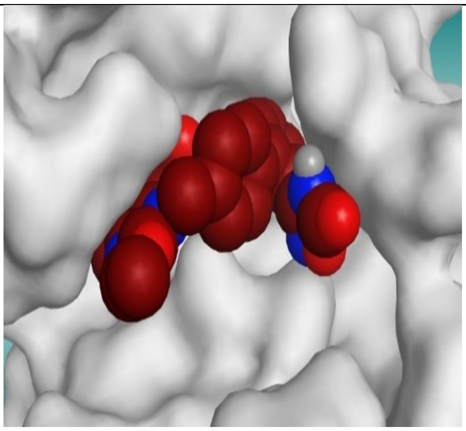	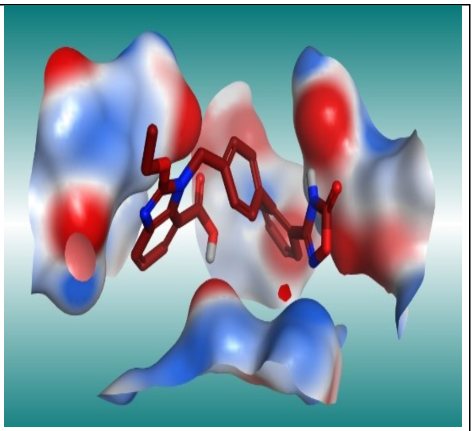
**10**	N3	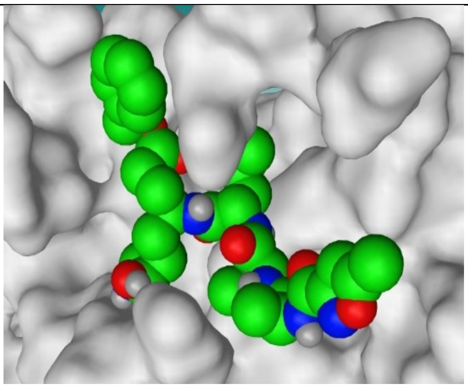	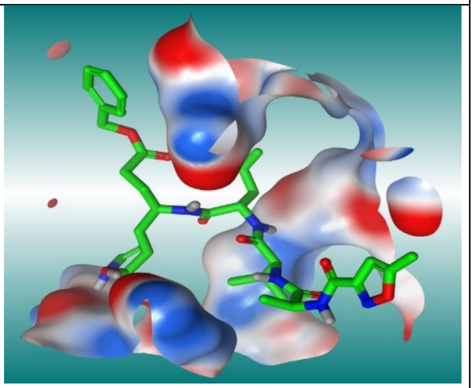
